# Prototyping a Versatile Two-Layer Multi-Channel Microfluidic Device for Direct-Contact Cell-Vessel Co-Culture

**DOI:** 10.3390/mi11010079

**Published:** 2020-01-10

**Authors:** Li-Jiun Chen, Bibek Raut, Nobuhiro Nagai, Toshiaki Abe, Hirokazu Kaji

**Affiliations:** 1 Department of Finemechanics, Graduate School of Engineering, Tohoku University, 6-6-01 Aramaki, Aoba-ku, Sendai 980-8579, Japan; 2 Division of Clinical Cell Therapy, United Centers for Advanced Research and Translational Medicine (ART), Tohoku University Graduate School of Medicine, 2-1 Seiryo, Aoba-ku, Sendai 980-8575, Japan; 3 Department of Biomedical Engineering, Graduate School of Biomedical Engineering, Tohoku University, 6-6-01 Aramaki, Aoba-ku, Sendai 980-8579, Japan

**Keywords:** microfluidics, microfabrication, organ-on-a-chip, trans-epithelial electrical resistance, multi-culture

## Abstract

Microfluidic devices are gaining increasing popularity due to their wide applications in various research areas. Herein, we propose a two-layer multi-channel microfluidic device allowing for direct-contact cell-vessel co-culture. Using the device, we built a co-culture model of the outer blood-retina barrier (oBRB), mimicking the in vivo retinal pigment epithelial cells-Bruch membrane-fenestrated choroids. To demonstrate the versatility of the design, we further modified the device by inserting platinum electrodes for trans-epithelial electrical resistance (TEER) measurement, demonstrating the feasibility of on-chip assessment of the epithelial barrier integrity. Our proposed design allows for direct-contact co-culture of cell–cell or cell–vessel, modifiable for real-time evaluation of the state of the epithelial monolayers.

## 1. Introduction

Organ-on-a-chip, a subset of lab-on-a-chip, is often associated with replicating tissue- or organ-level functions in integrated microfluidic devices to answer fundamental research questions such as cell physiology or to explore disease pathophysiology [1]. Several microfabrication techniques exist, which are used for different research applications, ranging from fabrication of biosensors and disease models to vascularization of engineering tissues for medical studies [2,3,4]. Narrowing down on medical applications, many in vitro models have been built utilizing bio-microelectromechanical systems (Bio-MEMS) or static transwell system to study different human organs. MEMS and microfluidic on-chip models have been covered and readers are referred to the reviews [1,3,4,5,6,7]. 

Focusing on microvessels and vasculature development, microfluidic models of microvessels and barrier properties have been extensively studied. Some of the models particularly focus on recapitulating the functionalities, i.e., the restrictive barrier properties or the responsiveness of the in vitro microvessels to exogenous stimulations. Wang et al. performed a tri-culture of pericytes, astrocytes, and endothelial cells inside microfluidic devices with embedded Ag/AgCl wire to characterize the restrictive blood–brain barrier (BBB) [8]. To study neuroinflammation of BBB, Herland et al. performed a tri-culture of astrocytes, pericytes, and endothelial cells in a single microchannel and subjected the model under tumor necrosis factor-alpha (a pro-inflammatory cytokine) [7]. Functional responses of the microvessel under stimulation (endothelial permeability and cytokine release) were analyzed. Using a similar approach, Price et al. tested the effect of mechanical factors on the restrictiveness of the endothelial barriers using a single microvessel [9]. On the other hand, multi-channel microfluidic devices that allowed examinations on the vascular formation and tracking morphology of microvessels over the course of experiments and under different stimulations have been developed and used in various applications [10,11,12,13,14].

**Figure 1:**
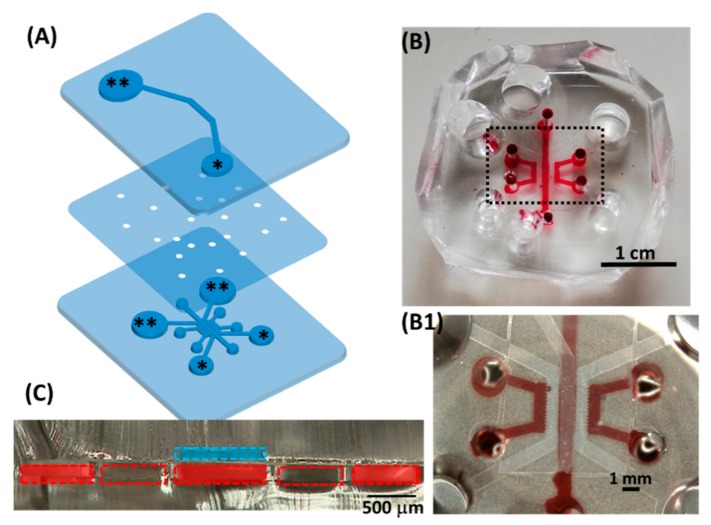
Configuration of the proposed device. (**A**) Exploded view; medium reservoirs are marked with asterisks (** as inlets, * as outlets when loading cells or media). (**B**) Top view of the assembled device, with dye water in some of the lower microchannels. (**B1**) Magnified view of the black dashed box in (**B**), showing the central part of the device overlaying the single top microchannel and multiple lower microchannels. (**C**) Microscopic cross-sectional view of the assembled two-layer device. The dashed blue line indicated the top microchannel; each dashed red box indicated the lower microfluidic channels separated by micropillars. Red rectangles in the lower channel corresponded to where red dye water was loaded. Together, the blue and red rectangles indicate the potential culturing chambers. The detailed dimension is in Figure A1.

Based on the pre-established design of a multichannel microfluidic device, we modified the device based on the Jeon research group [15] and the Kamm research group [16] into top-down, direct-contact cell–cell or cell–vascular co-culturing (Figure 1). Using the device, we built a model of the outer blood–retina barrier (oBRB), mimicking the in vivo retinal pigment epithelial cells-Bruch membrane-fenestrated choroids using human retinal pigmental epithelial cells (ARPE-19) and human umbilical vein endothelial cells (HUVEC). To demonstrate the versatility of the device, we slightly modified the design by embedding four Platinum electrodes (diameter 300 µm) for trans-epithelial electrical resistance (TEER) measurement. Using the modified device, we quantified the TEER of ARPE monolayers and the oBRB model. The device proposed in this study can be used as a multi-culturing cell and/or vessel model for various applications with the potential of connecting individual fluidic devices in a fluidic network, mimicking human physiology as demonstrated in the work of Edington et al. [17].

## 2. Materials and Methods

### 2.1. Cell Culture

GFP expressing human umbilical vein endothelial cells (GFP-HUVECs) were purchased from Angio-Proteomie (cAP-0001GFP, Boston, MA, USA) and routinely subcultured at 4 × 10^3^ cells/cm^2^ in 75-cm^2^ tissue culture-grade flasks coated with 0.1% gelatin. Human lung fibroblasts (NHLF) was purchased from Lonza (CC-2512, Basel, Switzerland) and subcultured based on the supplier’s protocol. Human retinal pigmented epithelial cells (ARPE-19) were kindly provided by Leonard Hjelmeland (Department of Ophthalmology, Section of Molecular and Cellular Biology, University of California, Davis, CA, USA) on 27 November 2000, and used after receiving approval from the Tohoku University Environmental & Safety Committee (no. 2014MdA-232-5). NHLF and ARPE-19 were cultured in Dulbecco’s Modified Eagle Medium (DMEM) supplemented with 10% fetal bovine serum (FBS, S1400-500, Biowest, France) and 1% Antibiotic-Antimycotic (100X, Gibco, 15240062) while GFP-HUVECs were cultured in Endothelial Growth Medium (Lonza CC-3162, Basel, Switzerland). All cultures were maintained at 37 °C in a humidified atmosphere containing 5% CO_2_. The passage number used for the experiments for each of the respective cell types was passage 20–30 for ARPE-19, passage 6–9 for NHLF, and passage 4–9 for GFP-HUVECs.

### 2.2. Fabrication of the Microfluidic Device

**Figure A1:**
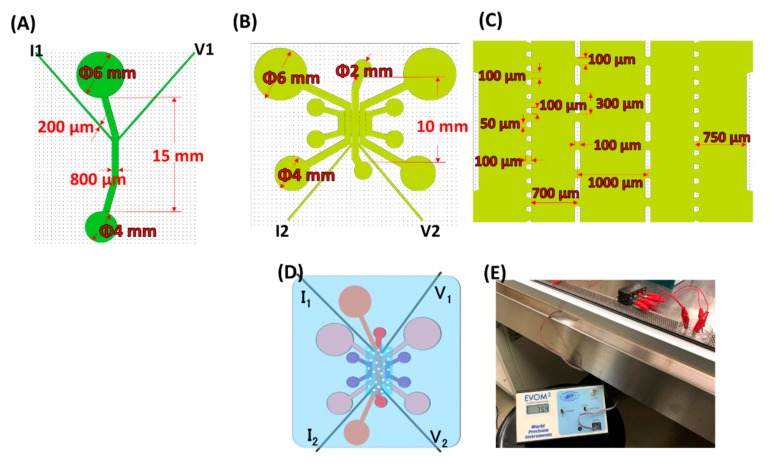
Dimensions of the microchannels for trans-epithelial electrical resistance (TEER) measurement. (**A**) Upper channel. (**B**) Lower channel. (**C**) Magnified view of (**B**), showing dimensions of multi-channels. (**D**) Top view of the assembled TEER device. (**E**) Experimental set-up for TEER measurement showing the connection between the microfluidic device and the EVOM instrument.

The microfluidic device used in the study has an upper microchannel and multiple lower microchannels, separated by a polyethylene terephthalate (PET) porous membrane of 8 µm in diameter (Falcon #353093). The device was made by conventional photolithography: the photomasks for the upper microchannel were designed in LayoutEditor (Justpertor GmbH, Unterhaching, Germany) and patterned by the Heiderlberg DWL66 laser writer. Thereafter, a master mold was made by spin-coating a 200 µm thick layer of SU-8 2100 (Microchem, Newton, MA, USA) on a silicon wafer and patterned by UV-lithography. Microchannels were made by liquid poly(dimethyl siloxane) (PDMS; Sylgard 184, Dow Corning, Midland, MI, USA) mixed at 10:1 ratio (wt/wt) of base polymer to curing agent, poured over the silicon master mold, degassed under vacuum pressure and cured in a 70 °C oven for at least 3 h. The cured PDMS was then cut into slabs and removed from the mold. The creation of the master mold and PDMS soft lithography for the lower microchannel followed the same procedure, except the lower PDMS slabs were made thin (2 mm in thickness) as opposed to the upper PDMS slabs. The thin lower PDMS slab was for microscopic observation while the upper PDMS slab was made thick to hold cell culturing media. The inlets and outlets of PDMS slabs were punched with the respective dimensions (Figure A1A,B), tapped to remove dust and impurities.

The membrane for the device was cut into 7 mm × 10.5 mm (width × length) out of the conventional PET membrane from Falcon transwell inserts (Falcon #353093). The membrane was bonded to the upper PDMS following the protocol developed previously [18]. After baking at 120 °C for an hour, the upper and lower channels treated with oxygen plasma were bonded. After a device was assembled, it was kept in a 120 °C oven for at least an hour. The bonding between the upper and lower PDMS slabs was checked (no leakage of ethanol) before use.

### 2.3. Design Prototype: Outer Blood–Retina Barrier Model

**Figure 2:**
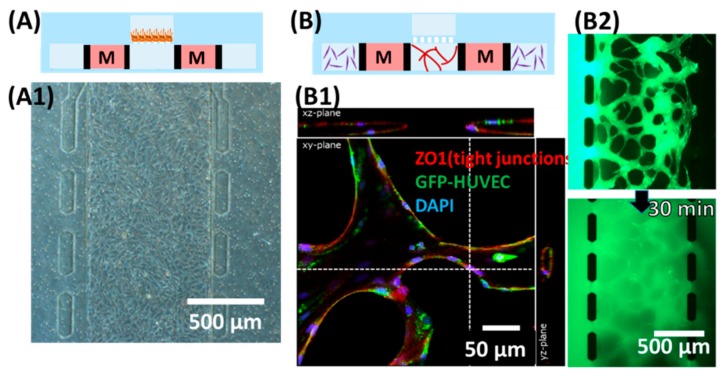
Demonstration of individual culturing chambers. (**A**) Culturing the human retinal pigmental epithelial cell (ARPE-19) monolayer in the upper channel. (**A1**) Microscopic view focusing on the upper channel, showing monolayer of ARPE. (**B**) Formation of microvascular lumens in the lower channels. “M” (medium channels) corresponded to * (lower channel) in Figure 1A. (**B1**) Confocal microscopic image staining for zonula occludens-1 (ZO1) and 4′,6-diamidino-2-phenylindole (DAPI). (**B2**) Snapshots taken after loading 70 kDa of FITC-dextran into one side of the lower channel immediately (**top**) and 30 min after (**bottom**).

**Figure 3:**
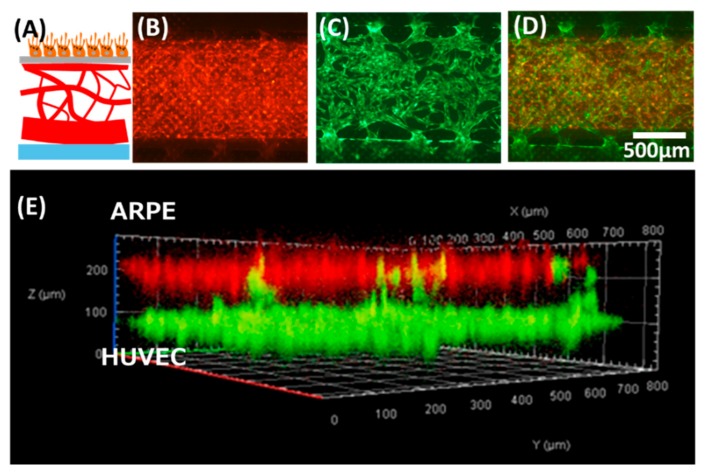
The oBRB model prototype built using the proposed device. (**A**) Schematic showing the anatomical structure of oBRB (retinal pigment epithelial cells–Bruch membrane-choroids). (**B**–**E**) tri-culture of ARPE–human umbilical vein endothelial cells (HUVEC)-human lung fibroblasts (NHLF). (**B**) ARPE cells stained in red were cultured in the upper channel. (**C**) GFP-HUVEC microvessels were cultured in the lower channel. (**D**) Overlay of (**B**) and (**C**). (**E**) Confocal microscopic view of the cross-section of the co-culture model.

Prior to seeding cells into microfluidic devices, 10 µg of fibronectin (Wako 063-05591) reconstituted in 1 mL PBS(-) filtered through 0.45 µm millipore was used to coat the upper microchannel (overnight in a 37 °C incubator) and washed with PBS-once before cell seeding. The formation of microvasculature was based on published work from previous studies [13,19]. Briefly, GFP-HUVECs and NHLF were detached from the culturing surfaces and made into suspensions of 7 × 10^6^ cells/mL. Fibrinogen precursor (Sigma-Aldrich F8630, St. Louis, MO, USA) 10 mg reconstituted in 1 mL of PBS(-) and filtered through 0.45 µm millipore was mixed with 3 times volume of GFP-HUVECs (at 7 × 10^6^ cells/mL) thoroughly. Thrombin (Sigma-Aldrich T7513, St. Louis, MO, USA) pre-aliquoted at 50 U/mL (two µL for 0.25 mg fibrinogen used) was mixed thoroughly with the cell–fibrinogen mixture which was immediately loaded into the central lower microchannel (Figure 1C, cross-section). The devices were kept in the 37 °C incubator for 5 min for the fibrin gel to polymerize. Thereafter, NHLF (at 7 × 10^6^ cells/mL) mixed with fibrinogen was loaded into both side channels (Figure 1C) following the same procedure, resulting in GFP-HUVECs and NHLF dispersed in 2.5 mg/mL fibrin (Figure 2B). After confirmation of the gelation, EGM supply was initiated by filling the inlet reservoirs (** in Figure 1A) and aspirating the medium from the outlets (* in Figure 1A) using a tip-cut 1 mL pipette. The microvasculature was cultured for 36 h before ARPE-19 were seeded, forming the proposed oBRB model (Figure 3). In seeding ARPE-19 into the upper channel, the medium in the upper channel was aspirated before cell seeding (at 10^7^ cells/mL, 10 µL per device). The devices were left in a 37 °C incubator for 2 h for ARPE-19 to attach, after which 100 µL of DMEM was supplied and aspirated from the other end of the upper channel to wash out unattached ARPE-19. The devices were then returned to the incubator for an additional 2 h before loading the medium. The culture media (DMEM for the upper channel, EGM for lower channels) were refreshed every 48 h.

### 2.4. Modification of the Device for Trans-Epithelial Electrical Resistance (TEER) Measurement

**Figure 4:**
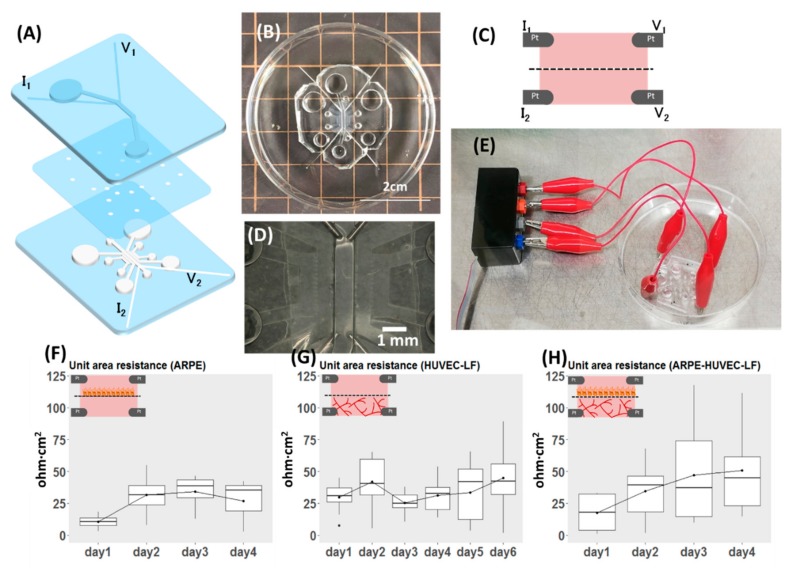
Demonstration of the versatility of the proposed design, with modification for measuring TEER. (**A**) Exploded view of the device. (**B**) Top view of the assembled device. (**C**) Schematic view of the cross-section showing the position of voltage and current electrodes. (**D**) Magnified view of (**B**) focusing on the position of electrodes inside the device. (**E**) Part of the system set-up showing the connection of the device to the electrode adapter, which is connected to the EVOM instrument for TEER measurement. (**F**–**H**) Results of TEER measurement reported as unit area resistance. The inset for each plot shows what was measured of (**F**) TEER of ARPE monolayer (n = 4). (**G**) TEER of co-culture of HUVEC and NHLF (n = 10). (**H**) TEER of the oBRB model (n = 11).

The masks of the upper and lower channels were redesigned with four microgrooves for placement of two electrodes in each of the upper and lower channels (Figure 4A, for dimensions, see Figure A1). The microgrooves for platinum (Pt) wire were designed and made at 200 µm in width, but Pt wire of 300 µm in diameter was used as it fits firmly inside the PDMS microgroove. In the last step of fabrication, four pieces of 3 cm long Pt wires were inserted into one device. The protrusion of wire inside the microchannel is approximately 1 mm (Figure 4D). After insertion, a drop of uncured PDMS was applied to the electrode channel’s entrance and cured to minimize the potential displacement of electrodes during handling alligator clippers for measurement (Figure 4E). Each microfluidic device underwent quality checks for the following: (1) porous membrane covered the entire lower microchannel length, except inlets and outlets, (2) the tip of an electrode protruded 1 mm into the microchannel, (3) the upper and the central lower channel were properly aligned. Devices that met the criteria were then kept sterilized until being used for experiments. The individual layer and the assembled device are shown in Figure 4.

### 2.5. TEER Measurement Setup

The Pt wires from the device were connected to four ports of an adaptor (Figure 4E) which was connected to the EVOM (World Precision Instruments, Sarasota, FL, USA) set to Ohm mode. To be consistent in the approach, the Pt wires were connected to current (I) and voltage (V) ports in the order shown in Figure 4A for all the devices. Due to variability in TEER readings between devices, the blank (acellular device filled with culture media: DMEM in the upper channel, EGM in the lower channel) for each sample was recorded before every experiment. Culture media were equilibrated to room temperature (kept 20 min in the clean bench) and readings were recorded in all cases to minimize variations in results.

### 2.6. Data Collection and Analysis

#### 2.6.1. Staining for Fluorescence Imaging

Microscopic images were captured using a fluorescent microscope (Olympus, Tokyo, Japan) or a confocal microscope (LSM700, Carl Zeiss MicroImaging, Gina, Germany). As part of the assessment of the growth of cells inside a microfluidic device, ARPE-19 cells were stained with Cell TrackerTM Fluorescent Probes red (5 µM, Molecular Probes) according to the manufacturer’s staining protocols. ARPE-19 were stained prior to seeding into devices.

#### 2.6.2. TEER Measurements

Samples (devices) used for experiments were individually labeled and the TEER readings and microvasculature morphology specific to each device were recorded. The number of samples is indicated in the corresponding figure captions. Due to the relatively small sample size, we did not test for the statistical significance of the difference in TEER readings between different days of culture. TEER values were extracted and reported as a unit area of resistance (ohm·cm^2^) by first subtracting the raw values from the blank and multiplied the resulting value by area in cm^2^. The area was calculated as width × length between electrodes (0.08 cm × 0.5 cm). Readings from gel-free, acellular devices were taken as the blank for the ARPE monolayers, while the blank for HUVEC-NHLF and ARPE-HUVEC-NHLF cultures was taken as the readings 30 min after seeding (i.e., cells embedded in fibrin gel) to take the effect of gel contributing TEER into account. The device was individually labeled and the blank and readings specific to each device were used in the calculation.

## 3. Results and Discussion

### 3.1. The Device as the Outer Blood–Retina Model

In building the model, we first evaluated the formation of ARPE monolayer in the device (Figure 2A). Separately, the formation of lumens of the HUVEC vasculature was confirmed by immunostaining of tight junction (Figure 2B1) and loading a diameter of 6 µm microbeads in one side of the inlet of the device (Video S1). Images of the microvascular network focused on a z-plane in Figure 2B2 were captured after flowing Fluorescein isothiocyanate (FITC)-dextran 70 kDa at 0.5 mg/mL in one side of the lower channel inlet. Figure 2B shows the schematic cross-sectional view of the multi-channel microfluidic device in which endothelial cells (HUVEC) and the supporting cells (NHLH) in two side channels were cultured (media channels were indicated M in Figure 2). After confirming the ability to culture epithelial cells and the vasculature, we attempted to demonstrate the feasibility of the multi-culture of cells and/or vessels using our proposed device and built a model of the outer blood–retina barrier (oBRB). The design of the microfluidic device was an extension based on previously developed models by Noo Li Jeon and Roger Kamm research groups where models have been used to study microvessels. Here, we further modified it to enable culturing of the ARPE-19 directly on the top of HUVEC microvasculature, mimicking oBRB where in vivo retinal pigment epithelial cells and the juxtaposed choriocapillaris are separated by the Bruch membrane (Figure 3). We adopted a larger pore size here as a demonstration of the model; the diameter of the pore, however, is customizable by using commercial membranes of different diameters. We had previously built the cell–cell oBRB model using ARPE-19 and HUVEC cells [20], characterizing the behavior of cells under pathological angiogenesis. We anticipate the prototype we proposed here to be used to characterize the epithelial monolayer microvasculature such as BRB or BBB, under pathological microenvironments.

### 3.2. Versatility of the Design: Modification for TEER Measurement

To demonstrate the versatility of our proposed microfluidic device, we modified it to allow for direct TEER measurement. TEER provides a quick, non-invasive, label-free and real-time indication of the state of cells as an alternative to perfusion of fluorescent-labeled molecules such as FITC-dextran in the evaluation of the barrier property. Both evaluation methods have their pros and cons: TEER offers an instant, real-time assessment of the state of cells while permeability assay is a simple yet elegant way that is typically used as an end-point assessment of the barrier integrity of a cell monolayer. In the designed device, two Pt wire in the upper channel corresponded for voltage and current measurements, the same configuration for the lower central channel (Figure 4A,C and Figure A1). The four-electrode measurement system was adopted here to be compatible with the EVOM instrument. The advantage of the four-electrode system is the negligible effect of double-layer capacitance, as the current supply and voltage measuring electrodes are separated [21]. As an alternative, the two-electrode measurement system can also be used here if we wish to take the impedance spectroscopy using a potentiostat. The two-layered microfluidic TEER device and the connection to the EVOM instrument are shown in Figure 4E and Figure A1E.

Many microfluidic TEER models had been proposed; a square culturing chamber was adopted by Helm et al. [22], while some measured the TEER of cells cultured in long microchannels [23,24]. Here, we further modified our device for TEER evaluation of a top-down cell-vessel model. We first measured the TEER of ARPE-19 monolayers and HUVEC-NHLF separately before conducting experiments on the oBRB models. TEER readings of ARPE monolayers (Figure 4F) increased by days after seeding, corresponding to the proliferation of cells inside the device. There was no obvious trend from the co-culture of HUVEC-NHLF (Figure 4G), due to the nature of the fenestrated vessels. We also evaluated the ARPE-19 of the oBRB model by measuring the TEER (Figure 4H), where the readings indicated the proliferation of ARPE-19. TEER values for HUVEC-NHLF culture and the oBRB model were higher than ARPE monolayer culture, perhaps due to the presence of fibrin gel and the influence of microvessels. The readings, however, were subjected to fluctuations arising from changes in temperature, and movement of electrodes during clipping. According to the datasheet of EVOM from World Precision Instruments, there is a 10% variability in TEER reading using the commercial STX2 chopstick electrodes on the transwell TEER measurement due to non-uniform current density flowing across the membrane, arising from variations in the electrode position. Such variation is expected to be even higher in the microfluidic system [25]. In our system, one of the possible causes of variations in reading between devices could be due to variations in the length of protrusion of electrodes inside the microchannel, although we attempted to keep it the same (1 mm) as much as possible. This can be further improved with micro-patterned electrodes. In our device, as the distance between measuring electrodes is 5 mm, we did not anticipate non-uniformity in the current density but suspected the fluctuations in readings as the result of differences in temperature when reading was taken [22].

### 3.3. Design of the Proposed Microfluidic Device

The feasibility of the proposed device as a platform for top-down multi-culture of cell-vessel was demonstrated by building a model of the human outer blood-retina barrier (oBRB). In addition, the versatility of the device was shown by modifying the device, allowing TEER evaluation of a cell monolayer. Although we only demonstrated the prototype of oBRB here, the device can be modified for specific organ-on-a-chip applications and studying physiological responses after taking allometric scaling and fluidic shear stress into account. Cell-vascular models are widely applicable to the human organ system, ranging from the blood–brain barrier (BBB) and glial interactions to interactions of trophoblasts and the blood vessels in the placenta. We have been developing integrated TEER measurement of the microfluidic system to evaluate the state of monolayer cells in real-time; such a system is applicable to evaluating the response of a monolayer to its environment, such as the BBB, the airway epithelium under shear stress, and mechanisms regulating intestinal epithelium. The model we proposed here is customizable and adaptable to different organs of origin, with potential extensibility for quantification of physical parameters such as barrier resistance and shear stress. We foresee that our proposed device has potential in disease modeling and drug testing.

## 4. Conclusions

We proposed a microfluidic device consisting of a single upper microchannel and multi-lower microchannels for direct-contact cell–vessel co-culture. The co-culture model was demonstrated by making the outer blood–retina barrier, made up of retinal pigment epithelial cells, endothelial cells, and the supporting cells. The versatility of the device was demonstrated by modifying the device for the insertion of Pt electrodes for TEER measurement. We measured the TEER of the ARPE monolayer and that of the oBRB model (ARPE–HUVEC–NHLF culture). Although only one aspect of the versatility design was demonstrated in this work, it is however, unlimited but extensible for investigation of the effect of shear stress on the multi-culture model by additionally gluing PDMS ports to the in/outlets of the device, or connect modular devices in an integrated fluidic network for studying physiological responses.

## Supplementary Materials

The following are available online at https://www.mdpi.com/2072-666X/11/1/79/s1, Video S1: Perfusion of microbeads demonstrating the formation of lumens.
